# Exploring the Molecular Mechanism of Tong Xie Yao Fang in Treating Ulcerative Colitis Using Network Pharmacology and Molecular Docking

**DOI:** 10.1155/2022/8141443

**Published:** 2022-09-27

**Authors:** Menglong Zou, Ying Zhu

**Affiliations:** The First Hospital of Hunan University of Chinese Medicine, Changsha, Hunan 410007, China

## Abstract

**Objective:**

The purpose of this study was to investigate the mechanisms of action of Tong Xie Yao Fang (TXYF) against ulcerative colitis (UC) by employing a network pharmacology approach.

**Methods:**

The network pharmacology approach, including screening of the active ingredients and targets, construction of the active ingredient-drug target network, the active ingredient-diseasetarget network, the protein–protein interaction (PPI) network, enrichment analyses, molecular docking, and targets validation, was used to explore the mechanisms of TXYF against UC.

**Results:**

34 active ingredients and 129 and 772 targets of TXYF and UC, respectively, were identified. The intersection of the active ingredient-drug target network, the active ingredient-disease target network, and the PPI network suggested that kaempferol, beta-sitosterol, wogonin, and naringenin were the core ingredients and prostaglandin-endoperoxide synthase 2 (PTGS2) was the core target. Enrichment analyses showed that regulation of exogenous protein binding and other functions were of great significance. Nuclear factor-kappa B (NF-*κ*B) signaling pathway, interleukin-17 (IL-17) signaling pathway, and tumor necrosis factor (TNF) signaling pathway were important pathways. Results of molecular docking indicated that the core ingredients and the target molecule had strong binding affinities. We have validated the high levels of expression of PTGS2 in UC by analyzing three additional datasets from the Gene Expression Omnibus (GEO) database.

**Conclusions:**

There are multiple ingredients, targets, and pathways involved in TXYF's effectiveness against UC, and these findings will promote further research and clinical applications.

## 1. Introduction

Ulcerative colitis (UC) is a chronic nonspecific inflammatory bowel disease with diffuse inflammation of both the colon and rectum mucosa [1]. The disease is also associated with abdominal pain, diarrhea, hematochezia, and systemic symptoms of varying severity [1]. Globally, there are estimated to be 8 million cases of UC, and rates are on the rise [2]. A hospital stay is required in approximately 20% of UC patients, and up to 10% are eventually required to undergo colectomy surgery [3, 4]. Additionally, people with UC have a higher incidence of colorectal cancer than people without [5]. Because of this, UC has a major impact on both mind and body. While medicinal treatments, such as 5-ASA and corticosteroids, are effective treatments for UC, their side effects limit their use [6, 7]. It is therefore necessary to find other alternative treatment options for UC. The use of traditional Chinese medicine (TCM) has been around for thousands of years to treat refractory and common diseases [8]. Its validity, however, is often questioned since there is a lack of high-level evidence [9]. TCM has multiple ingredients and targets, and there is some evidence that some Chinese medicines improve the intestinal functional barrier in UC [10].

Tong Xie Yao Fang (TXYF) is a classic and famous Chinese medicine prescription [11]. Four herbs (Table 1) are included in TXYF, which were first described in a Yuan dynasty book named “Dan Brook Heart Law.” It has been used for hundreds of years to treat diarrhea and abdominal pain associated with UC [12]. Previously, we reviewed the mechanisms of action of these treatments, including anti-inflammatory and immunomodulatory effects, regulation of intestinal flora and the brain-gut axis, and promotion of mucosal healing [11]. The results of clinical studies also show that TXYF is effective and has few adverse effects in the treatment of UC [13]. There is, however, still much to learn about its molecular mechanisms.

In treating diseases, herbs contain many ingredients and target multiple targets, making it difficult to identify their pharmacodynamic ingredients and molecular mechanisms. With the advancement of network technology, the combination of network pharmacology, molecular docking, and bioinformation provides a powerful method for uncovering the complex molecular mechanisms of TCM [14]. They increase the likelihood of discovering the best drug candidates and significantly cut down on the initial time and expense of conducting experiments to determine drug-target interactions [15–17]. Thus, the goal of this study was to elucidate the potential mechanism by which TXYF against UC, using a network pharmacological strategy and molecular docking.

## 2. Materials and Methods

### 2.1. Screening the Active Ingredients of TXYF

“Baizhu,” “Baishao,” “Chenpi,” and “Fangfeng” were used as search terms in the Traditional Chinese Medicine System Pharmacology Database (TCMSP, https://tcmspw.com/tcmsp.php) [18] and the Encyclopaedia of Traditional Chinese Medicine (ETCM, https://www.tcmip.cn/ETCM/) [19] to acquire related ingredients, respectively. The ingredients meeting both bioavailability (OB) ≥ 30% and drug-likeness (DL) ≥ 0.18 were screened out as the active ingredients [20].

### 2.2. Predicting the Targets of the Active Ingredients

We extracted the corresponding targets of the active ingredients of TXYF from the TCMSP database. To make the targets of TXYF more comprehensive, we retrieved the SymMap database (https://www.symmap.org/) [21] to add descriptions of target information. And then we converted the targets to gene symbols by using UniProt KB (https://www.uniprot.org/) [22]. Nonhuman genes were removed. Moreover, theingredients corresponding to nonhuman gene also were deleted.

### 2.3. Active Ingredient-Drug Target Network Construction

A network of active ingredient-drug target was created to visualize the complex pharmacodynamics relationships between herbs of TXYF by the employment of Cytoscape v3.8.0. In Cytoscape, we used the plug-in cytoNCA to calculate the value of degree centrality (DC) and closeness centrality (CC) of all nodes in the network. As a standard for filtering ingredients and targets, we considered the nodes with values greater than the median value of DC and CC as the key nodes in our network [23]. Based on the new network, we can intuitively identify relationships between TXYF's active ingredients and its target targets.

### 2.4. Seeking Out Disease-Related Targets

From the Gene Expression Omnibus (GEO, https://www.ncbi.nlm.nih.gov/geo/) [24], study number GSE65114 gene expression profile chip data have been gathered. The chip platform was GPL16686. This data set consists of mucosal biopsy specimens collected from 16 patients with UC and 12 healthy people. Identifying differential genes based on these criteria: |log FC| > 1.5, *p* < 0.05.

### 2.5. Active Ingredient-Disease Target Network Construction

Targets from TXYF and UC were intersected to determine intersections. Cytoscape software was used to visualize the active ingredient-disease target network based on the active ingredients of TXYF and the intersection targets.

### 2.6. Protein–Protein Interaction (PPI) Network Construction

PPI network was built by importing the intersection targets into the STRING database (https://www.string-db.org/) [25], and the result was exported in tab-separated value (TSV) format. Subsequently, PPI data were visualized with Cytoscape software.

### 2.7. Enrichment Analysis

Using R software, we performed enrichment analysis on the target genes of TXYF in the treatment of UC based on Gene Ontology (GO) and Kyoto Encyclopaedia of Genes and Genomes (KEGG).

### 2.8. Molecular Docking

To determine what is the core ingredients of TXYF in the treatment of UC, we intersected steps 2.3 and 2.5. In the PubChem database (https://pubchem.ncbi.nlm.nih.gov/) [26], we entered the core ingredients to obtain small molecule ligand structures. Likewise, we intersected steps 2.3 and 2.6 to identify the core targets of TXYF in the treatment of UC. The core target proteins were entered into the RCSB PDB database (https://www.rcsb.org/) [27] and the 3D structure was downloaded. Molecular docking was performed with AutoDockTools software after preparing the ligand files and the receptor file. Docking modules are considered more stable when their binding energy is smaller [28]. In terms of minimum binding energy, a value lower than −5.0 kcal/mol indicates a good affinity between receptor and ligand, and a value lower than −7.0 kcal/mol indicates a very strong affinity between receptor and ligand [29, 30]. To better evaluate the docking result, we also downloaded mesalazine-D3 (CID: 71750020) from PubChem database as ligand to perform molecular docking. Visualization of all docking results was performed with PyMOL v2.1.4 software [31].

### 2.9. Target Validation

Three additional data sets were retrieved from GEO for validation. This includes GSE36807 (7 healthy controls and 15 UC), GSE38713 (13 healthy controls and 22 UC), and GSE10616 (11 healthy controls and 10 UC). These three validation data sets were then used to verify the expression levels of the core targets.

## 3. Results and Analysis

### 3.1. Active Ingredients and Targets of TXYF

**Figure 1:**
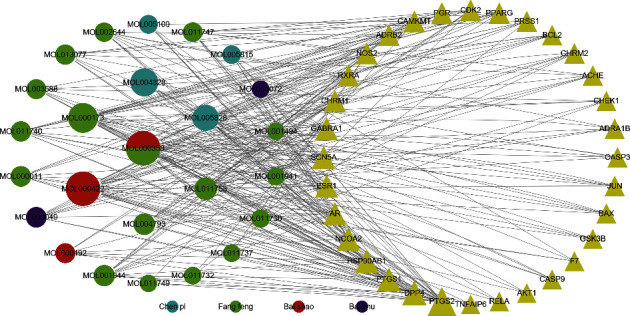
The network diagram of active ingredient-drug target. The circle nodes represent ingredients, and the targets are indicated by triangle nodes. Node size is proportional to its DC value.

We obtained 108 active ingredients (Table 2) of TXYF in the TCMSP database and the ETCM database that satisfy both screening factors of OB ≥ 30% and DL ≥ 0.18 simultaneously. Among them, there are 35 ingredients for Baishao, 16 ingredients for Baizhu, 31 ingredients for Chenpi, and 26 ingredients for Fangfeng. A total of 34 active ingredients of TXYF were obtained after deleting 40 repeated ingredients and 34 ingredients corresponding to nonhuman gene. We identified 129 targets corresponding to 34 active ingredients in TXYF by searching TCMSP database and SymMap database. The Cytoscape software was used to draw a network diagram of the 34 active ingredients and the 129 drug targets. Next, we used the CytoNCA for further analysis of the topology of the network. The median value of DC and CC of the network was 2 and 0.320158103, respectively. According to the median value of BC and DC, 57 nodes were further screened (Figure 1).

### 3.2. Differential Gene in UC

**Figure 2:**
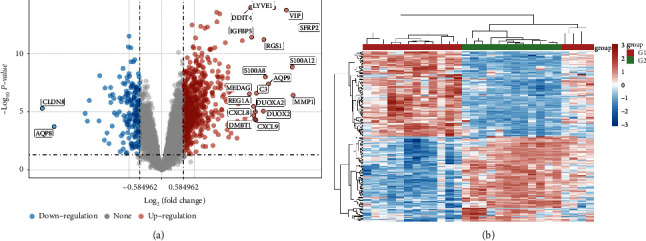
(a) Volcano map. (b) Heatmap. *Note*. G1, mucosal biopsy specimens of healthy people; G2, mucosal biopsy specimens from patients with UC; red, high gene expression; blue, low gene expression.

GSE65114 data was analyzed using R software for screening differential genes for UC. 772 differential genes were screened according to screening criteria, 607 of which were upregulated and 165 were downregulated (Figure 2(a)). The first 50 differentially expressed genes of UC mucosal biopsy specimens and healthy mucosal biopsy specimens are selected, and a differentially expressed gene heatmap is drawn using the R software. Gene expression levels are represented by the color of the heatmap: blue represents decrease, red represents increase, and increasing and decreasing degrees of gene expression are represented by the brightness of the color, as shown in Figure 2(b).

### 3.3. Active Ingredient-Disease Target Network Construction

**Figure 3:**
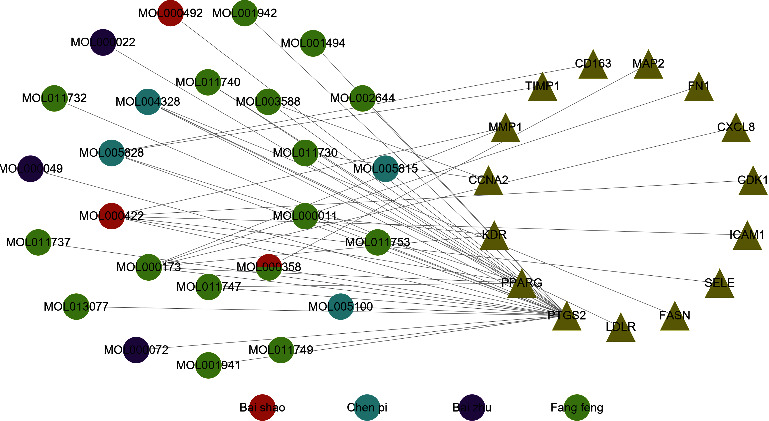
Active ingredient-disease target network. Circle nodes represent active ingredients in TXYF, triangle nodes represent overlapping gene symbols between disease and drug, with edges indicating that nodes can interact.

The 129 drug gene symbols and the 772 disease gene symbols were intersected to find 15 common targets. Using these 15 common targets, we constructed an active ingredient-disease target network. The network shown in Figure 3 illustrates the complex relationship between TXYF and UC.

### 3.4. PPI Network Construction

**Figure 4:**
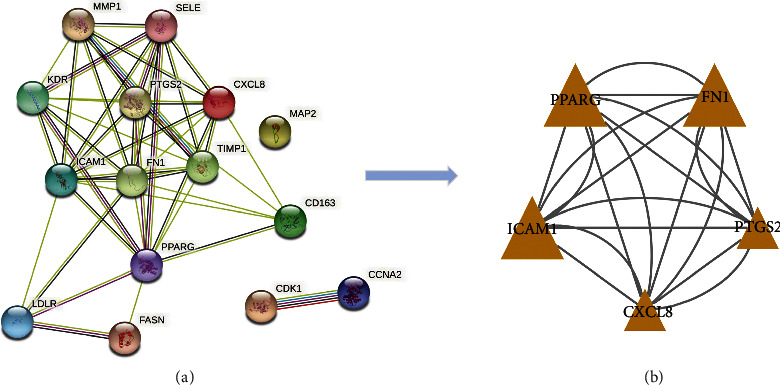
(a) The PPI network obtained from the STRING database platform. (b) The PPI network that extracted genes with value greater than the median value of DC and CC.

STRING platform provided us with a map of the action relationships between 15 overlapping genes (Figure 4(a)). We exported the PPI network in “TSV format,” and then analyzed its topological properties using Cytoscape software. The median value of DC and CC of the network is 16 and 0.30952381, respectively. We then selected all genes with values greater than the median value of DC and CC and generated 5 targets of more significant networks (Figure 4(b)).

### 3.5. GO Enrichment Analysis

**Figure 5:**
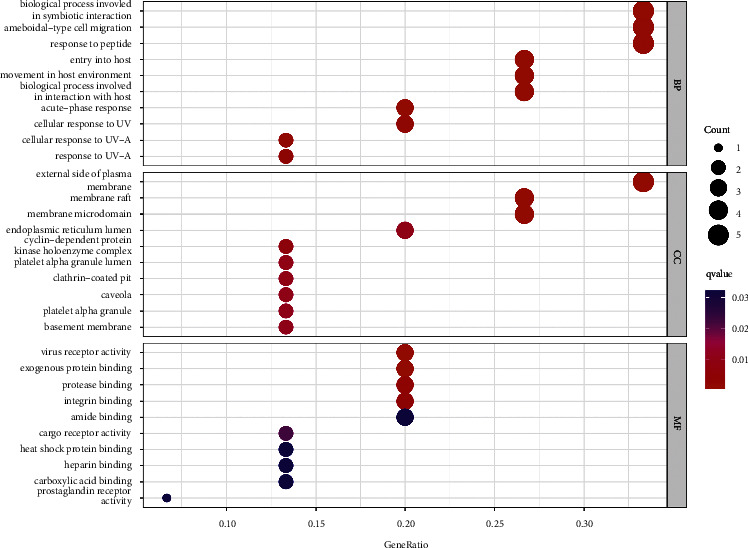
GO enrichment analysis of the overlapping targets.

15 overlapping genes were categorized and enriched according to three modules by GO enrichment analysis (*p* < 0.05 and *q* < 0.05): biological process (BP), molecular function (MF), and cellular component (CC), with 751 GO terms enriched, of which the proportion of BP terms was relatively high, with 650 GO terms, mainly showing how some proteins interact with biological pathways and how they transport, such as BP involved in symbiotic interaction (GO: 0044403), entry into host (GO: 0044409), acute-phase response (GO: 0006953), and movement in host environment (GO: 0052126). 35 GO terms are present in CC, and these overlapping genes are closely related to those cell biofilms, like external side of plasma membrane (GO: 0009897), membrane raft (GO: 0045121), and membrane microdomain (GO: 0098857). The overlapping genes enriched 66 GO terms for MF, and MF analysis contributed to understanding which receptor activities the overlapping genes influence as well as how partial protein binding occurs, mainly virus receptor activity (GO: 0001618), exogenous protein binding (GO: 0140272), and protease binding (GO: 0002020).  Figure 5 shows *q* value intercepts for the top ten terms from small to large for an abbreviated presentation of GO enrichment results.

### 3.6. KEGG Enrichment Analysis

**Figure 6:**
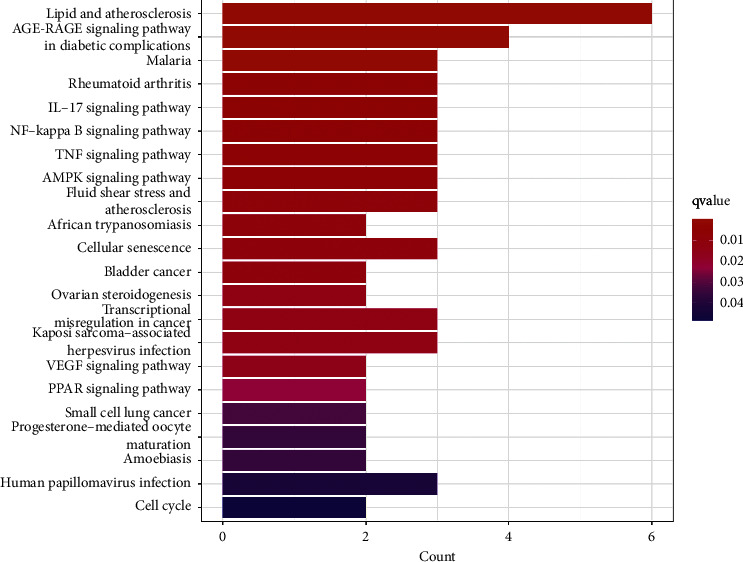
KEGG enrichment analysis of overlapping targets.

R software was used to perform the KEGG enrichment analysis, with screening conditions set at *p* < 0.05 and *q* < 0.05. As illustrated in Figure 6, TXYF is mainly involved in treating UC through the lipid and atherosclerosis (hsa05417), the IL-17 signaling pathway (hsa04657), the NF-kappa B signaling pathway (hsa04064), the TNF signaling pathway (hsa04668), the AMPK signaling pathway (hsa04152), and the PPAR signaling pathway (hsa03320).

### 3.7. Molecular Docking

**Figure 7:**
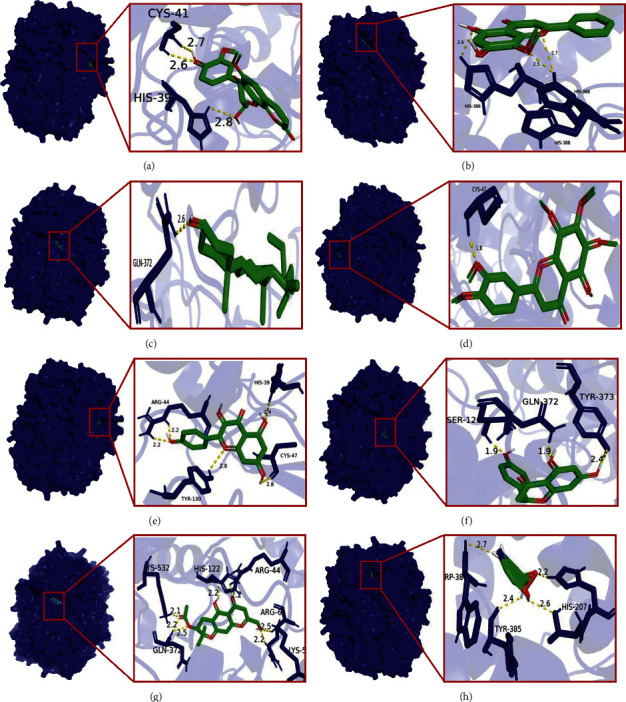
The core ingredients' interactions with PTGS2. (a) MOL000011 with PTGS2, (b) MOL000173 with PTGS2, (c) MOL000358 with PTGS2, (d) MOL005828 with PTGS2, (e) MOL000422 with PTGS2, (f) MOL004328 with PTGS2, (g) MOL011740 with PTGS2, and (h) mesalazine-D3 with PTGS2.

Based on the active ingredient-drug target network diagram (Figure 1), MOL000358, MOL000422, MOL000173, MOL004328, MOL005828, MOL011753, MOL000011, MOL000049, MOL013077, and MOL011740 may contribute to the pharmacodynamic material basis of TXYF. As well, PTGS2, DPP4, NCOA2, HSP90AB1, SCN5A, GABRA1, AR, ESR1, and NOS2 may be the ten most important targets of TXYF used to treat diseases. Figure 3 shows that MOL000173, MOL000422, MOL005828, MOL004328, MOL011740, MOL000358, MOL011730, MOL000011, MOL000358, and MOL011747 may be ten of the important active ingredients of TXYF against UC. The PPI network diagram (Figure 4(b)) indicates that TXYF may target 5 highly important targets in the treatment of UC: ICAM1, PPARG, FN1, PTGS2, and CXCL8. Besides the interactions between drugs and diseases, we must also consider the effects of the drugs themselves. Hence, we obtained 1 core target (PTGS2) by taking the intersection of 10 important targets in the active ingredient-drug target network and 5 important targets in the PPI network. Likewise, we obtained 7 core ingredients (MOL011740, MOL000358, MOL000422, MOL000173, MOL004328, MOL005828, and MOL000011) by taking the intersection of 10 important ingredients in the active ingredient-drug target network and 10 important ingredients in the active ingredient-diseases target network. Notably, the core target and core ingredients obtained here were screened according to their topological parameters. Based on the published literature and preliminary information in the TCMSP database, we investigated whether the core ingredients directly interact with the core target. All the core ingredients can directly affect the core target, which is interesting. Molecular docking is a methodology for predicting drug and protein binding modes and binding affinity based on receptor characteristics [45]. A ligand's conformational stability to the receptor is determined by its binding energy: lower binding energy means higher stabilization [29, 30]. After performing the analysis described above and predicting the results, we selected 7 core ingredients in TXYF to dock with PTGS2 to validate the results (Figure 7). As well, we downloaded mesalazine-D3's two-dimensional structure from the PubChem database for molecular docking to compare with TXYF's seven core ingredients (Figure 7). Based on the results, all the core ingredients exhibited strong binding affinity to the core target (Table 3). (2R, 3R)-3-(4-hydroxy-3-methoxy-phenyl)-5-methoxy-2-methylol-2,3-dihydropyrano[5,6-h][1,4]benzodioxin-9-one (MOL000011) and kaempferol (MOL000422) showed the smallest binding energy and considered as the most effective ingredients against PTGS2 protein. The largest binding energy was observed for mesalazine-D3 with −7.1 kcal/mol.

### 3.8. Validation of the Core Target

**Figure 8:**
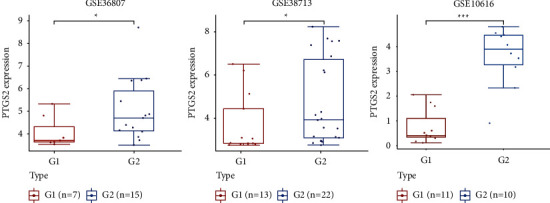
Validation of PTGS2 expression in UC patients. *Note*. Boxplot shows PTGS2 expression in mucosal biopsy specimens of patients with UC and healthy people in three GEO datasets (GSE36807, GSE38713, and GSE10616). G1, mucosal biopsy specimens of healthy people; G2, mucosal biopsy specimens from patients with UC. *Note*. ^*∗*^<0.05, ^∗∗^<0.01, ^∗∗∗^<0.001.

Three independent datasets (GSE36807, GSE38713, and GSE10616) were analyzed for PTGS2 expression data to validate the core target's expression. In the validation datasets, PTGS2 was similarly upregulated with statistical significance in UC (Figure 8). Therefore, PTGS2 may be a promising therapeutic target for TXYF treatment of UC.

## 4. Discussion

TCM has been used for thousands of years to treat various diseases. There are many classical prescriptions recorded in TCM literature that are still used in China today with clinical effectiveness. TXYF, one of classical TCM prescriptions, is composed of *Atractylodes macrocephala* Koidz (Baizhu), Paeoniae Radix Alba (Baishao), *Citrus Reticulata* (Chenpi), and *Saposhnikoviae* Radix (Fangfeng) [46]. Our study explored possible mechanisms of TXYF related to UC using network pharmacology and molecular docking, which indicated that 7 ingredients and 15 target genes, especially PTGS2 (prostaglandin-endoperoxide synthase 2), were related to UC. According to the results of GO and KEGG pathway enrichment analyses, we found that the effects of the TXYF against UC may be due to the core ingredients of the TXYF, especially for kaempferol (MOL000422) and (2R,3R)-3-(4-hydroxy-3-methoxy-phenyl)-5-methoxy-2-methylol-2,3-dihydropyrano[5,6-h][1,4]benzodioxin-9-one (MOL000011) which could influence the regulation of the NF-*κ*B (nuclear factor-kappa B), IL-17 (interleukin-17), and TNF (tumor necrosis factor) signaling pathway. They may also be related to virus receptor activity, movement in host environment, exogenous protein binding, and protease binding. PTGS2 was selected as a core target based on the results of the PPI network analysis and the active ingredient-drug target network analysis, and it shows a greater affinity with the core ingredients than mesalazine-D3 based on molecular docking results.

The etiology and mechanisms of UC are complex [47]. Animal and clinical studies have confirmed the potential of herbal immunomodulators in UC [48–50]. There is evidence that mucosal immune cells produce inflammation factors that play an essential role in the pathogenesis of UC [51]. Active macrophages produce proinflammatory cytokines such as IL-17 and TNF-*α*, which can aggravate UC [52]. A balance between pro- and anti-inflammatory cytokines is essential to the control of UC [53]. In UC, anti-TNF induces mucosal healing, which reflects TNF's important role in pathogenesis [54]. A multitude of evidence points to the NF-*κ*B pathway as having a vital role in the pathogenesis of UC [55, 56]. NF-*κ*B overexpression in mucosal macrophages causes the production of proinflammatory cytokines, which harms mucosal tissues [57]. It has been shown that TXYF treatment reduces expression of NF-*κ*B p65 gene and protein in rats, indicating that TXYF prevents overactivation of the NF-*κ*B pathway [58]. Proinflammatory and anti-inflammatory factors are regulated by TXYF, which inhibits the expression of proinflammatory promoters and increases the expression of anti-inflammatory inhibitors [58]. In a study of 62 patients with UC, the control group was given sulfasalazine and the treatment group was given a combination of TXYF and sulfasalazine. One month after treatment, IL-17 and interferon-*γ* levels decreased statistically in the treatment group compared with the control group [59]. By soothing the liver and strengthening the spleen, TXYF may have an overall regulating effect, thereby repairing colon tissue, inhibiting the expression of TNF-*α*, and eliminating inflammation [60]. In our study, the NF-*κ*B, IL-17 and the TNF signaling pathways are key pathways involved treatment of TXYF in UC.

PTGS2, also known as cyclooxygenase-2 (COX-2), encodes a prostaglandin synthase that catalyzes the synthesis of arachidonic acid derivatives (prostaglandins) [61]. In colonic epithelia of UC mice, mTOR complex 1 (mTORC1) was hyperactive [62]. It was found that colonic epithelial TSC1 (mTORC1 negative regulator) disruption increased the level of mTORC1 activity in the colon epithelia and aggravated UC [63]. Importantly, COX-2 inhibitor reversed elevated proinflammatory mediator levels caused by TSC1 deficiency, and subsequently mice models of UC were reduced in symptom severity and pathological characteristics [64]. When the prostaglandin E receptor 2 (PGE2) signaling pathway synergizes with TNF-*α*, it increases TNF-*α*-induced inflammatory responses, thereby escalating PG-mediated inflammation [65]. Reducing inflammation may be a key mechanism for treating UC, and anti-inflammatory agents may prove useful in preventing UC. A study conducted by Guo and Yan [66] revealed that TXYF can promote ulcer healing in rats with UC by downregulating the expression of COX-2 in the colonic mucosa, which is consistent with our results. It is widely known that LPS can trigger the release of inflammatory mediators like COX-2, IL-6, and IL-8. A study conducted by Wang et al. [67] showed wogonin inhibited the expression of COX-2 and restricted the translocation of NF-*κ*B to the nucleus, thereby maintaining intestinal barrier integrity in LPS-inducedCaco-2 cells. *β*-sitosterol is a common sterol found in herbal medicines. Lee et al. [68] found that *β*-sitosterol improved the colon mucosa barrier by suppressing the expression of inflammatory factors TNF-*α* and COX-2, as well as the activation of NF-*κ*B. The flavonoid kaempferol has been shown to have anti-inflammatory properties as well as to be immune-modulating [69]. Several structural changes were observed in colonic samples from DSS-induced mice, including mucosal ulcerations, crypt destruction, and loss of goblet cells [70]. On the other hand, histological analysis of the colons of mice treated with kaempferol revealed reduced levels of tissue damage [71]. In UC model, cytokines are controlled by many cells involved in inflammation, such as macrophages, neutrophils, and others. Myeloperoxidase (MPO) reflects neutrophil recruitment. When compared to other flavonols, Regasini et al. [72] found that kaempferol derivatives significantly reduced MPO activity. Similarly, Kanashiro et al. [73] showed kaempferol inhibited human neutrophil degranulation significantly. The increased levels of COX-2-derived PGE2 are found at sites of intestinal inflammation and correlate with disease activity, exactly as described previously. It was reported by Park et al. [74] that consumption of kaempferol-supplemented diets are able to significantly lower colonic COX-2 mRNA expression. Likewise, nobiletin inhibited inflammation by downregulating the expression of COX-2 and inducible nitric oxide synthase (iNOS) in colitic rats [75]. In our study, we preliminarily explored the mechanism of the TXYF in treating UC by using network pharmacology and molecular docking. NF-*κ*B, IL-17, and TNF signaling pathways are closely regulated by kaempferol, (2R,3R)-3-(4-hydroxy-3-methoxy-phenyl)-5-methoxy-2-methylol-2,3-dihydropyrano[5,6-h][1,4] benzodioxin-9-one, nobiletin, and divaricatol, and so on, according to our findings. PTGS2 was found to be the core target of the TXYF in treating UC and it reflects a strong affinity with the core ingredients, such as aempferol, and so on.

Nonetheless, some limitations were present in our study. In the evaluations, the ingredients and targets were derived mostly from databases. However, some ingredients and targets may have been omitted. Moreover, dose of herbs was not taken into consideration. Further experimentation is therefore needed to verify TXYF's multiple mechanisms of action for treating UC, both in vivo and in vitro.

## 5. Conclusion

We obtained the core active ingredients of TXYF in the treatment of UC, namely, kaempferol, (2R,3R)-3-(4-hydroxy-3-methoxy-phenyl)-5-methoxy-2-methylol-2,3-dihydropyrano[5,6-h][1,4]benzodioxin-9-one, nobiletin, divaricatol, beta-sitosterol, wogonin, and naringenin. By constructing the active ingredient-drug target network and PPI network, 1 core gene was screened, namely, PTGS2. GO functional enrichment analysis showed that the cross gene mainly performed exogenous protein binding and other functions, and the results of KEGG pathway enrichment analysis showed that the cross gene was mainly involved in NF-*κ*B, IL-17, TNF, and other signaling pathways. Finally, molecular docking is vital to testing and exploring the binding between active ingredients and targets.

## Figures and Tables

**Table 1:** The formulation of Tong Xie Yao Fang (one dose).

Herb		Medicinal part	Amount in application (g)
Latin name	Chinese name		
*Atractylodes macrocephala* Koidz	Bai zhu	Rhizomes	12
Paeoniae Radix Alba	Bai shao	Root	12
*Citrus Reticulata*	Chen pi	Peel	9
*Saposhnikoviae* Radix	Fang feng	Root	6

**Table 2:** Active ingredients of TXYF.

No.	Drug	Molecule ID	Ingredient name	OB	DL	Reference
1	Fangfeng	MOL011737	Divaricatacid	87.00	0.32	[32]
2	Fangfeng	MOL004793	Marmesine	84.77	0.18	[32]
3	Fangfeng	MOL000392	Formononetin	69.67	0.21	
4	Fangfeng	MOL000011	(2R,3R)-3-(4-hydroxy-3-methoxy-phenyl)-5-methoxy-2-methylol-2,3-dihydropyrano[5,6-h][1,4]benzodioxin-9-one	68.83	0.66	[32]
5	Fangfeng	MOL005429	Hancinol	64.01	0.37	[33]
6	Fangfeng	MOL011732	Anomalin	59.65	0.66	[33]
7	Fangfeng	MOL004792	Nodakenin	57.12	0.69	[33]
8	Fangfeng	MOL001944	Marmesin	50.28	0.18	[33]
9	Fangfeng	MOL011730	11-hydroxy-sec-o-beta-d-glucosylhamaudol_qt	50.24	0.27	[32]
10	Fangfeng	MOL000417	Calycosin	47.75	0.24	[33]
11	Fangfeng	MOL002392	Deltoin	46.69	0.37	[32]
12	Fangfeng	MOL001942	Isoimperatorin	45.46	0.23	[34]
13	Fangfeng	MOL011749	Phelloptorin	43.39	0.28	[32]
14	Fangfeng	MOL001494	Mandenol	42.00	0.19	[34]
15	Fangfeng	MOL002644	Phellopterin	40.19	0.28	[34]
16	Fangfeng	MOL007514	Methyl icosa-11,14-dienoate	39.67	0.23	[34]
17	Fangfeng	MOL013077	Decursin	39.27	0.38	[34]
18	Fangfeng	MOL011753	5-O-methylvisamminol	37.99	0.25	[34]
19	Fangfeng	MOL000358	Beta-sitosterol	36.91	0.75	[32]
20	Fangfeng	MOL000359	Sitosterol	36.91	0.75	[32]
21	Fangfeng	MOL000358	Beta-sitosterol	36.91	0.75	[32]
22	Fangfeng	MOL003588	Prangenidin	36.31	0.22	[32]
23	Fangfeng	MOL001941	Ammidin	34.55	0.22	[32]
24	Fangfeng	MOL011747	Ledebouriellol	32.05	0.51	[32]
25	Fangfeng	MOL011740	Divaricatol	31.65	0.38	[34]
26	Fangfeng	MOL000173	Wogonin	30.68	0.23	[32]
27	Chenpi	MOL011737	Divaricatacid	87.00	0.32	[35]
28	Chenpi	MOL005815	Citromitin	86.90	0.51	[35]
29	Chenpi	MOL004793	Marmesine	84.77	0.18	[35]
30	Chenpi	MOL000392	Formononetin	69.67	0.21	[35]
31	Chenpi	MOL000011	(2R,3R)-3-(4-hydroxy-3-methoxy-phenyl)-5-methoxy-2-methylol-2,3-dihydropyrano[5,6-H][1,4]benzodioxin-9-one	68.83	0.66	[36]
32	Chenpi	MOL005429	Hancinol	64.01	0.37	[35]
33	Chenpi	MOL005828	Nobiletin	61.67	0.52	[35]
34	Chenpi	MOL011732	Anomalin	59.65	0.66	[37]
35	Chenpi	MOL004328	Naringenin	59.29	0.21	[35]
36	Chenpi	MOL004792	Nodakenin	57.12	0.69	[38]
37	Chenpi	MOL001924	Paeoniflorin	53.87	0.79	[38]
38	Chenpi	MOL001944	Marmesin	50.28	0.18	[37]
39	Chenpi	MOL011730	11-hydroxy-sec-O-beta-D-glucosylhamaudol_Qt	50.24	0.27	[36]
40	Chenpi	MOL000417	Calycosin	47.75	0.24	[38]
41	Chenpi	MOL005100	5,7-dihydroxy-2-(3-hydroxy-4-methoxyphenyl)chroman-4-one	47.74	0.27	[36]
42	Chenpi	MOL002392	Deltoin	46.69	0.37	[37]
43	Chenpi	MOL001942	Isoimperatorin	45.46	0.23	[35]
44	Chenpi	MOL011749	Phelloptorin	43.39	0.28	[37]
45	Chenpi	MOL001494	Mandenol	42.00	0.19	[35]
46	Chenpi	MOL002644	Phellopterin	40.19	0.28	[37]
47	Chenpi	MOL007514	Methyl icosa-11,14-dienoate	39.67	0.23	[38]
48	Chenpi	MOL013077	Decursin	39.27	0.38	[38]
49	Chenpi	MOL011753	5-O-methylvisamminol	37.99	0.25	[37]
50	Chenpi	MOL000359	Sitosterol	36.91	0.75	[35]
51	Chenpi	MOL000358	Beta-sitosterol	36.91	0.75	[35]
52	Chenpi	MOL000359	Sitosterol	36.91	0.75	[35]
53	Chenpi	MOL003588	Prangenidin	36.31	0.22	[37]
54	Chenpi	MOL001941	Ammidin	34.55	0.22	[37]
55	Chenpi	MOL011747	Ledebouriellol	32.05	0.51	[37]
56	Chenpi	MOL011740	Divaricatol	31.65	0.38	[37]
57	Chenpi	MOL000173	Wogonin	30.68	0.23	[37]
58	Baizhu	MOL009431	Stemonine	81.75	0.72	[39]
59	Baizhu	MOL000392	Formononetin	69.67	0.21	[39]
60	Baizhu	MOL000022	14-acetyl-12-senecioyl-2E,8Z,10E-atractylentriol	63.37	0.3	[40]
61	Baizhu	MOL000020	12-senecioyl-2E,8E,10E-atractylentriol	62.40	0.22	[40]
62	Baizhu	MOL000021	14-acetyl-12-senecioyl-2E,8E,10E-atractylentriol	60.31	0.31	[40]
63	Baizhu	MOL005360	Malkangunin	57.71	0.63	[39]
64	Baizhu	MOL005384	Suchilactone	57.52	0.56	[40]
65	Baizhu	MOL000049	3*β*-acetoxyatractylone	54.07	0.22	[40]
66	Baizhu	MOL009154	Tuberostemoenone	53.90	0.73	[40]
67	Baizhu	MOL009387	Didehydrotuberostemonine	51.91	0.74	[40]
68	Baizhu	MOL000028	*α*-Amyrin	39.51	0.76	[40]
69	Baizhu	MOL009436	Stemotinine	38.69	0.46	[40]
70	Baizhu	MOL000358	Beta-sitosterol	36.91	0.75	[40]
71	Baizhu	MOL000033	(3S,8S,9S,10R,13R,14S,17R)-10,13-dimethyl-17-[(2R,5S)-5-propan-2-yloctan-2-yl]-2,3,4,7,8,9,11,12,14,15,16,17-dodecahydro-1h-cyclopenta[a]phenanthren-3-ol	36.23	0.78	[41]
72	Baizhu	MOL000072	8*β*-ethoxy atractylenolide III	35.95	0.21	[40]
73	Baizhu	MOL009361	13,15-dideoxyaconitine	34.67	0.25	[41]
74	Baishao	MOL001918	Paeoniflorgenone	87.59	0.37	[42]
75	Baishao	MOL003958	Evodiamine	86.02	0.64	[42]
76	Baishao	MOL004793	Marmesine	84.77	0.18	[42]
77	Baishao	MOL001925	paeoniflorin_qt	68.18	0.4	[42]
78	Baishao	MOL001928	albiflorin_qt	66.64	0.33	[42]
79	Baishao	MOL007016	Paeoniflorigenone	65.33	0.37	[42]
80	Baishao	MOL001910	11alpha,12alpha-epoxy-3beta-23-dihydroxy-30-norolean-20-en-28,12beta-olide	64.77	0.38	[42]
81	Baishao	MOL000785	Palmatine	64.60	0.65	[42]
82	Baishao	MOL005807	Sen-byakangelicol	58.00	0.61	[43]
83	Baishao	MOL004792	Nodakenin	57.12	0.69	[43]
84	Baishao	MOL000211	Mairin	55.38	0.78	[43]
85	Baishao	MOL000492	(+)-catechin	54.83	0.24	[42]
86	Baishao	MOL001924	Paeoniflorin	53.87	0.79	[42]
87	Baishao	MOL001924	Paeoniflorin	53.87	0.79	[43]
88	Baishao	MOL001944	Marmesin	50.28	0.18	[43]
89	Baishao	MOL000096	(−)-Catechin	49.68	0.24	[42]
90	Baishao	MOL001921	Lactiflorin	49.12	0.8	[42]
91	Baishao	MOL002710	Pyrethrin Ii	48.36	0.35	
92	Baishao	MOL001942	Isoimperatorin	45.46	0.23	[42]
93	Baishao	MOL001919	(3S,5R,8R,9R,10S,14S)-3,17-dihydroxy-4,4,8,10,14-pentamethyl-2,3,5,6,7,9-hexahydro-1h-cyclopenta[a]phenanthrene-15,16-dione	43.56	0.53	[44]
94	Baishao	MOL000422	Kaempferol	41.88	0.24	[42]
95	Baishao	MOL002662	Rutaecarpine	40.30	0.6	[43]
96	Baishao	MOL002644	Phellopterin	40.19	0.28	[42]
97	Baishao	MOL002464	1-Monolinolein	37.18	0.3	[44]
98	Baishao	MOL007061	Methylenetanshinquinone	37.07	0.36	[44]
99	Baishao	MOL000359	Sitosterol	36.91	0.75	[42]
100	Baishao	MOL000358	Beta-sitosterol	36.91	0.75	[42]
101	Baishao	MOL000359	Sitosterol	36.91	0.75	[42]
102	Baishao	MOL000358	Beta-sitosterol	36.91	0.75	[42]
103	Baishao	MOL001454	Berberine	36.86	0.78	[42]
104	Baishao	MOL006346	Itraconazole	36.67	0.33	[43]
105	Baishao	MOL005789	Neobyakangelico L	36.18	0.31	[43]
106	Baishao	MOL001939	Alloisoimperatorin	34.80	0.22	[42]
107	Baishao	MOL001930	Benzoyl paeoniflorin	31.27	0.75	[42]
108	Baishao	MOL007025	Isobenzoylpaeoniflorin	31.14	0.54	[42]

**Table 3:** Results of molecular docking.

Target	Binding energy (kcal/mol)							
	MOL000011	MOL000173	MOL000358	MOL005828	MOL000422	MOL004328	MOL011740	Mesalazine-D3
PTGS2	−9.0	−8.7	−8.5	−8.4	−9.0	−8.0	−0.84	−7.1

## Data Availability

Specific study data are available from the corresponding author upon request.
